# Aging, Poverty, and Healthcare Access and Affordability in Nigeria: Implications for Policy

**DOI:** 10.1002/puh2.70125

**Published:** 2025-09-15

**Authors:** Sunkanmi Folorunsho

**Affiliations:** ^1^ Department of Sociology University of Nebraska‐Lincoln Lincoln Nebraska USA

**Keywords:** aging, healthcare, older adults, policy, poverty

## Abstract

**Background:**

Nigeria's aging population is expanding rapidly. Older adults face intersecting challenges of poverty, chronic disease burden, and inadequate access to healthcare. With limited formal income support and minimal health insurance coverage, most elderly Nigerians rely on family or continue informal labor to survive. This compounds their vulnerability in later life.

**Objectives:**

This article examines the economic and health‐related barriers to healthcare utilization among older Nigerians. It incorporates recent demographic and epidemiological trends, wealth‐based inequalities, rural–urban disparities, and evolving policy responses. The analysis integrates insights from the Nigeria Demographic and Health Survey (NDHS) 2018 and applies Andersen's healthcare access model.

**Methods:**

A perspective approach was adopted to synthesize empirical literature, national data (including NDHS), and recent policy developments such as the National Health Insurance Authority Act and the National Senior Citizens Centre (NSCC) Act. Comparisons are drawn with other lower‐middle‐income countries, notably India, to highlight global relevance.

**Findings:**

Older Nigerians, particularly women and rural dwellers, experience high poverty rates, with up to 85% of women aged 70 and above living in poverty. They face chronic multimorbidity and have some of the lowest health service utilization rates due to cost, distance, and systemic neglect. Although policy frameworks, such as the NSCC and Basic Health Care Provision Fund (BHCPF), exist, implementation remains weak.

**Conclusions:**

Comprehensive reforms are essential to improve elderly health outcomes in Nigeria. Priorities include expanding subsidized health insurance, implementing universal social pensions, strengthening rural health services, and combating ageism. A coordinated and inclusive policy strategy can transform aging from a crisis into an opportunity for national development.

## 1 Introduction

Globally, the older adult population is rapidly growing, and with this demographic shift comes a heightened demand for healthcare [1]. According to the United Nations, by 2050 the number of people aged 60 years and above is projected to more than double, reaching 2.1 billion worldwide [2]. In developing countries like Nigeria, where poverty is widespread, the challenges of aging are especially pronounced as limited resources constrain access to essential services [3]. The World Health Organization (WHO) defines healthy aging as maintaining functional ability and well‐being in older age [3]. However, achieving healthy aging is more difficult in Nigeria due to widespread poverty and geographical barriers, particularly in rural and underserved areas [3, 4].

As Nigeria's population grows older, a significant portion of older adults face challenges in accessing healthcare—especially those living in poverty [4]. The intersection of aging, poverty, and limited healthcare access is a critical concern in Nigeria's health system [5]. Limited financial resources and underdeveloped health infrastructure disproportionately affect older Nigerians, who often have greater healthcare needs [6]. This perspective explores the links between aging and poverty in Nigeria, highlights barriers to healthcare accessibility and affordability, discusses the health challenges of aging, and examines existing policies. Finally, it outlines policy reforms aimed at improving the well‐being of Nigeria's aging population. Nigerian policymakers must address these multifaceted challenges through evidence‐based interventions to improve healthcare access and quality of life for older adults.

## 2 Trends and Patterns of Age–Sex Structure in Nigeria

Nigeria's population age structure is youthful, but the absolute number of older adults is increasing steadily [7]. The share of Nigerians over age 65 has remained around 3% of the total population for decades [7], reflecting high fertility and a large base of younger people. However, because Nigeria is so populous, it already has the highest number of older people in Africa [8]. In 2019, an estimated 8.2 million Nigerians were aged 60 or above; this number is projected to increase to about 25 million by 2050 [8]. In other words, the older adult population is expected to roughly triple by mid‐century, even though it will still be a relatively small fraction of Nigeria's much larger population growth [8]. This impending growth of the older cohort is an important demographic trend that will strain social support systems and healthcare services in the near future [8].

The age–sex distribution of Nigeria's older population shows that women tend to outnumber men at older ages, due to women's slightly higher life expectancy and survival rates [9]. Life expectancy in Nigeria remains low (about 60 years for men and 64 for women in 2020), but those who reach old age often live with chronic health conditions [9]. Most of Nigeria's older adults still reside in rural areas, as many retirees return to villages or never migrate to cities [10]. This means rural communities are bearing the brunt of population aging, despite having fewer resources. Overall, Nigeria's demographic pattern is one of a still youthful nation with a small but rapidly growing older adult segment [10]. Planning for this shift is crucial, as the country will need to support tens of millions of older citizens within a few decades. A solid understanding of the population's age–sex structure and its trajectory provides an important foundation for addressing the challenges discussed next.

## 3 Epidemiological Transition in Nigeria

Nigeria is undergoing an epidemiological transition in which patterns of disease change with aging. Younger segments of the population continue to face infectious and tropical diseases—malaria, tuberculosis, diarrheal disease, and other communicable illnesses remain major causes of morbidity and mortality [11]. However, as Nigerians reach middle and older ages, noncommunicable diseases (NCDs) become more prevalent. Older adults in Nigeria increasingly suffer from chronic conditions such as hypertension, diabetes, cardiovascular diseases (like stroke), arthritis, and impaired sensory function [12]. For instance, metabolic and cardiovascular risk factors (high blood pressure, obesity, etc.) are far more common in older age groups than among the young [13]. These NCDs now account for a growing share of deaths in Nigeria's older population, similar to patterns observed in other countries undergoing demographic transition [11].

At the same time, Nigeria's epidemiological transition is incomplete, and many older Nigerians still contend with communicable diseases and poor living conditions. Infectious diseases can severely affect seniors whose immunity is weakened by age or malnutrition. For example, pneumonia and malaria continue to take a toll on older people in Nigeria, whereas in more developed countries, these are less common causes of older adult illness [14]. Compared to a country like India, Nigeria's health profile for older adults is not vastly different in kind—Indian seniors also face rising rates of chronic diseases alongside persistent infectious disease challenges. In both countries, rapid urbanization and lifestyle changes contribute to increased NCD prevalence, and healthcare systems struggle to manage the dual burden [14]. The key difference is that India has implemented some nationwide health programs for NCDs and older adult care, whereas Nigeria's healthcare system has been slower to adapt [3]. In essence, as Nigerians age, they experience a shift from predominantly infectious disease burdens to predominantly chronic disease burdens, all compounded by poverty and limited access to care [15]. This transition points to the need for a healthcare system that can address chronic disease management (such as long‐term treatment of diabetes or heart disease) while still controlling infectious diseases in this vulnerable group [15].

## 4 The Link Between Aging and Poverty in Nigeria

Aging and poverty in Nigeria are deeply interconnected, with older adults disproportionately affected by economic hardship [16, 17]. Many older Nigerians, particularly in rural areas, have little or no access to formal employment income after they stop working. Without pensions or savings, they often become financially vulnerable in old age. In the absence of adequate pension coverage, most older individuals must rely on their families or continue working in informal jobs to survive [18]. In essence, evidence suggests that about 70% of older Nigerians are vulnerable and poor, unable to meet daily financial, nutritional, or healthcare needs [19]. Older people in Nigeria—especially those in rural areas—have the greatest risk of living in poverty due to frail health, low earnings, and limited economic opportunities [20]. Many rural older adults continue labor‐intensive work (such as subsistence farming or petty trading) well into old age because they cannot afford to retire, which further strains their health [21]. This cycle of lifelong poverty contributes to reduced access to basic services and poorer health outcomes among Nigeria's seniors.

Nigeria's pension system, intended to provide financial security for retirees, has largely been ineffective for the majority of older adults. The contributory pension scheme (established in 2004 and expanded in 2014) mainly covers formal sector workers, who make up a small fraction of the labor force [22]. Pensioners in the public system frequently experience delays in receiving payments or never receive their full entitlements at all [22]. As a result, even many retired civil servants struggle to cover basic living expenses, including medical bills. For the vast majority who worked in the informal sector, there is essentially no pension income. Nigeria's pension assets amount to only about 6%–7% of GDP and effectively cater to just 3% of the older adult population [23]. The absence of a social safety net or universal pension means that most older Nigerians have no guaranteed income. Some older adults are fortunate to be supported by financially secure children or relatives, but such informal support is inconsistent and often insufficient [24]. The traditional family support system in Nigeria is under strain as younger generations migrate to cities or abroad for work, leaving fewer resources for parents back home [25]. Consequently, many seniors find themselves in precarious economic situations.

Older individuals in rural Nigeria face additional challenges that compound their poverty. Many rural seniors never held formal employment and must keep working in physically demanding roles (like farming or manual labor) despite advancing age [26]. Rural areas also typically lack banking services and credit facilities, limiting the ability of older adults to secure any financial cushion. Additionally, healthcare and social services are scarce in rural regions, forcing older people to travel long distances (at significant cost) to access government pensions or medical care [27]. These hardships underline the urban–rural disparity: Urban older adults might have somewhat better access to clinics or charitable services, whereas rural older adults are often isolated with neither services nor support.

Table 1 summarizes indicators of the economic vulnerability of Nigeria's older population. It highlights the high incidence of poverty and low coverage of formal support systems among older adults.

**TABLE 1:** Economic vulnerability of older Nigerians (aged 60).

Indicator	Estimated value	Source
a. Older people classified as poor or economically vulnerable	Approximately 70%–75%	[28]
b. Older people who are severely poor or deprived	27% overall (36% of women; 20% of men)	[19]
c. Older individuals (65+) with access to formal pension income	Approximately 3%	[2]
d. Older population residing in rural areas	Approximately 64%	[29]

These statistics illustrate the precarious economic position of Nigeria's aging population. A large majority of older Nigerians experience poverty across multiple dimensions (income, living conditions, and health), and older women are especially vulnerable due to factors like lower lifetime earnings and widowhood [19]. Only a tiny minority of seniors receive reliable pension incomes, reflecting the narrow coverage of the formal pension scheme. The rest must depend on family support or personal labor. This high level of poverty directly translates into reduced ability to afford healthcare, as discussed in the next section.

## 5 Healthcare Access and Affordability for Older Adults in Nigeria

Older adults in Nigeria face major barriers to healthcare, especially due to cost and geographic inaccessibility [11]. Most health services are paid out‐of‐pocket because insurance coverage is limited and rarely includes older people [20]. In urban areas, hospitals and clinics may be physically accessible, but high costs make care unaffordable for many seniors [21]. Chronic conditions, such as hypertension or arthritis, require regular treatment, yet the cost of doctor visits, tests, and medications is often prohibitive. Economic reforms in the 1980s increased privatization and introduced user fees in public facilities, pushing many older adults to delay or skip needed care, which worsens health outcomes [28]. Additionally, in rural areas, the situation is even more challenging. Health centers are few, often under‐resourced, and located far from residents. Many older adults cannot travel long distances due to mobility limitations or lack of transportation [30]. Most primary care centers are not equipped to handle geriatric needs, so seniors often resort to traditional medicine or home remedies. Poverty, distance, and inadequate infrastructure leave many rural elders without access to basic healthcare [29].

**FIGURE 1:**
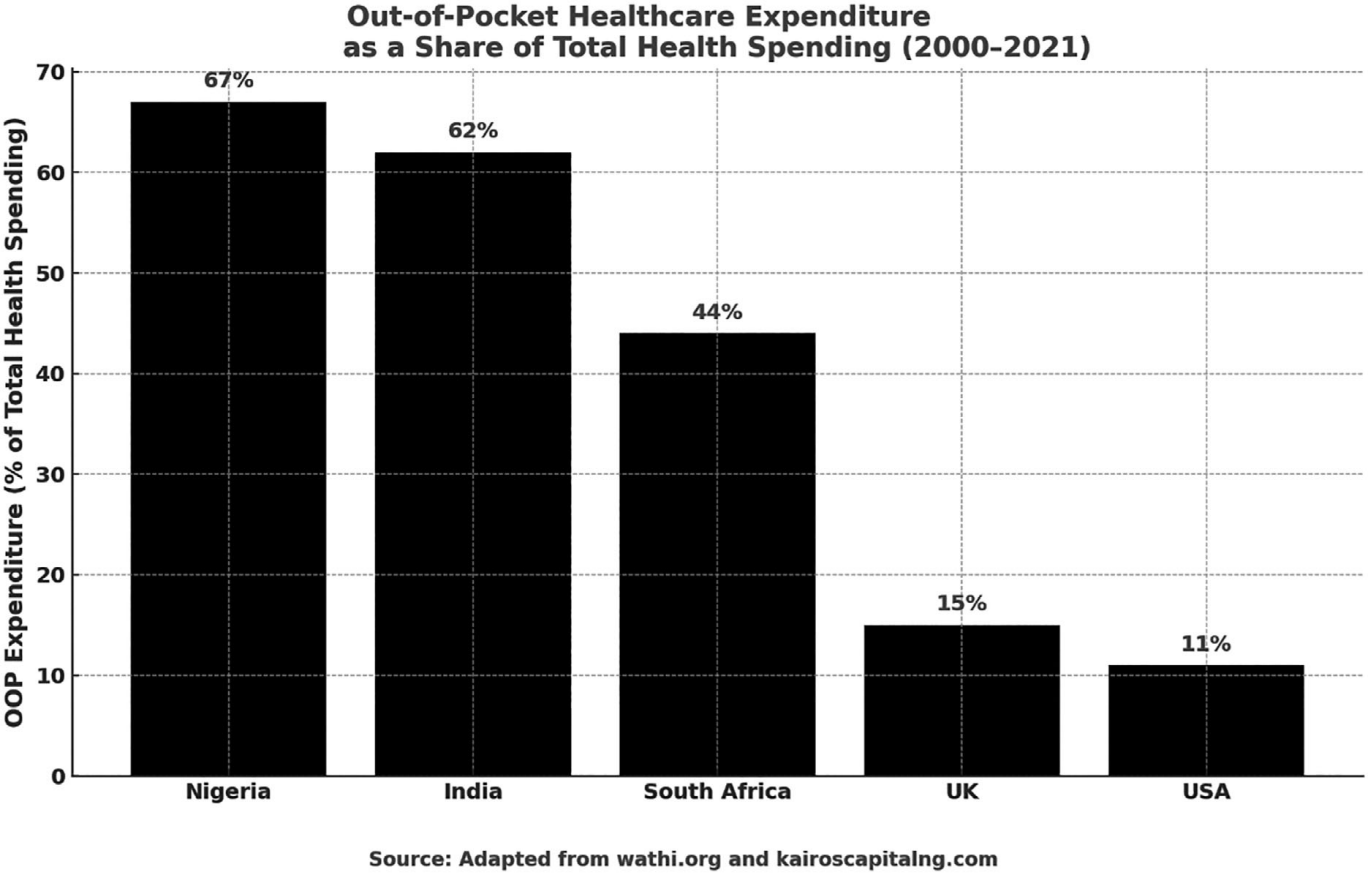
Out‐of‐pocket spending in Nigeria compared to other countries.

Urban–rural and income inequalities exacerbate the problem. Wealthier or urban seniors are more likely to access private healthcare, whereas poorer and rural elders struggle to receive even basic services. As shown in Figure 1 below, Nigeria's healthcare financing is among the most regressive in the world, with 60%–70% of health expenditures paid out‐of‐pocket between 2000 and 2021 [18].

High out‐of‐pocket costs mean that many seniors cannot afford essential treatments for chronic diseases. With limited income in old age, older adults often forgo or delay care, leading to poorer health outcomes. Families bear the financial burden, and in low‐income households this can deepen poverty [30]. As a result, poor older Nigerians, particularly in rural areas, experience significantly worse health outcomes than their wealthier peers. Even in cities, older adults with limited income cannot afford private care. In rural areas, services are often unavailable [29]. Decades of underinvestment in the public health system have reduced the quality and affordability of care. Without financial protection or nearby services, many older Nigerians are left with no choice but to rely on home treatments or unregulated providers. This contributes to growing health inequities driven by aging and poverty.

## 6 Existing Policies and Programs for Older Adult Health in Nigeria

Nigeria has begun to acknowledge the growing needs of its aging population through the development of policies and programs, though most remain limited in scope or early in implementation, and their impact on improving older adults’ well‐being is yet to be fully realized [31]. A significant milestone was the approval of the National Policy on Aging in 2021, which provides a framework for elderly care. This led to the establishment of the National Senior Citizens Centre (NSCC) through the NSCC Act of 2017, tasked with coordinating programs and identifying the needs of older adults. The NSCC is currently working on a dedicated health insurance scheme for senior citizens in partnership with stakeholders, but the initiative remains in the planning stages [32]. A key challenge is identifying sustainable funding sources.

The National Health Insurance Scheme (NHIS), launched in 2005 to promote universal health coverage, has had limited success [32]. Fewer than 5% of Nigerians are currently covered [33]. The scheme mainly benefits formal sector workers, thereby excluding the majority of older Nigerians, especially informal workers and rural dwellers [33]. Although provisions for voluntary or informal sector participation exist, uptake has been minimal due to poor awareness and affordability. A 2022 reform through the National Health Insurance Authority Act made insurance mandatory and established a Vulnerable Group Fund to subsidize premiums for the poor, including older adults [34]. However, as of 2025, implementation remains a significant challenge. Additionally, some public health programs have aimed to serve vulnerable populations. The 2014 National Health Act introduced the Basic Health Care Provision Fund (BHCPF), which allocates federal resources to deliver free primary healthcare to vulnerable groups, including poor older adults. Select states have leveraged the BHCPF to waive fees at primary health centers for registered low‐income households [35]. For example, a local government in Enugu State launched a mobile healthcare program offering check‐ups, medications, and referrals for seniors aged 60 and above. Additionally, NGOs have conducted free outreach clinics offering essential drugs for chronic diseases like hypertension and diabetes. Although promising, these initiatives remain localized and unsystematic [36].

Regarding social protection, Nigeria lacks a universal, noncontributory pension scheme. A notable pilot occurred between 2012 and 2014 in Ekiti State, where poor older residents received a stipend through a donor‐supported program [37]. Although effective, this initiative was short‐lived and not scaled nationally. The current contributory pension scheme, administered by the National Pension Commission, covers only formal sector workers, about 11 million people, leaving the vast majority of Nigeria's estimated 30 million adults aged 50 and above without pension benefits [29]. Moreover, recent policy revisions signal growing awareness. The draft National Social Protection Policy includes proposals to classify older adults as a vulnerable group eligible for cash transfers and healthcare subsidies [38]. Advocacy organizations, such as the Coalition of Societies for the Rights of Older Persons in Nigeria (COSROPIN), continue to push for legislation to strengthen seniors’ rights and welfare. Relevant bills are currently under consideration in the National Assembly [38].

## 7 Policy Implications and Recommendations

To address the complex challenges facing Nigeria's aging population, the country must implement comprehensive, multi‐sectoral reforms across health, social protection, and community development [9]. A critical starting point is ensuring that existing health insurance frameworks, such as those established under the National Health Insurance Authority Act (2022), are effectively implemented and expanded to provide comprehensive coverage for older adults and other vulnerable populations. Although the Act, along with provisions like the BHCPF from the 2014 National Health Act, offers a foundation for universal coverage and free primary healthcare for vulnerable groups, these mechanisms have yet to reach most poor older Nigerians due to slow rollout, inadequate funding, and limited awareness. Strengthening the implementation of these existing policies, alongside targeted enrollment and subsidy strategies, would significantly reduce financial barriers and improve access to care for older adults. Specific provisions must be made to enroll adults aged 60 and above, especially those living below the poverty line, through full subsidies funded by mechanisms such as the Vulnerable Group Fund [7]. Ensuring comprehensive coverage for common age‐related conditions such as cataracts, hypertension, diabetes, and arthritis would reduce financial barriers, improve preventive care, and enhance health outcomes. Experiences from countries like South Africa and Ghana demonstrate that social health insurance can significantly benefit older populations when implemented effectively.

Equally important is the need to strengthen and decentralize healthcare services, particularly in rural and underserved areas where the majority of Nigeria's older adults reside. Rural health facilities are often ill‐equipped and understaffed, and many older adults face physical and financial barriers to accessing distant urban hospitals [31, 32]. Investment is needed to upgrade primary care centers, train providers in geriatric care, and ensure the availability of essential medications. Mobile clinics and home‐based outreach services can extend care to immobile or remote seniors, whereas transportation support programs such as ambulance services or travel stipends can further reduce access challenges [14]. Empowering state and local governments to develop localized responses, supported by targeted funding from national initiatives like the BHCPF, is crucial to bridging the urban–rural divide in health access.

Beyond healthcare, Nigeria must address the severe economic vulnerability of its older population. The current pension system excludes the majority of older adults, especially those who worked in the informal sector [15]. Expanding contributory pension schemes through micro‐pension initiatives and introducing a modest, universal social pension for adults above a designated age, such as 70, would provide a financial safety net for those most in need. Ensuring timely and transparent disbursement of pension payments is equally essential [12]. Complementary interventions, such as targeted cash transfers or utility subsidies for older adults in low‐income households, can further reduce poverty and improve quality of life.

Social inclusion and community‐based support structures also play a vital role in older adult well‐being. Community health workers and volunteers should be trained to check on isolated or home‐bound seniors and provide basic health education and referrals. Government partnerships with NGOs, religious groups, and local associations can support the development of senior centers, social clubs, and day programs that foster engagement and reduce loneliness [39]. Public awareness campaigns should promote positive images of aging, counter ageist stereotypes and reinforce cultural values of respect and care for elders. Furthermore, geriatric training should be integrated into the curricula of medical and nursing schools to build a healthcare workforce better equipped to meet the unique needs of older populations.

Finally, attention must be given to family caregivers and older individuals living with disabilities. Most care for older adults in Nigeria is provided informally by family members, who often face emotional, physical, and financial strain [36]. Policies should recognize and support these caregivers through training, respite care services, and financial incentives. For seniors with disabilities, such as those affected by stroke, visual impairment, or mobility limitations, efforts should focus on improving accessibility in healthcare facilities and providing assistive devices such as eyeglasses, hearing aids, and walking aids. The overarching principle guiding all reforms should be to promote aging with dignity, ensuring that every older Nigerian, regardless of income, location, or physical ability, receives the respect and support they deserve.

## 8 Conclusion

Nigeria's aging population faces significant and multifaceted challenges related to poverty, healthcare access, and social well‐being. Currently, the country's economic and healthcare systems are not adequately equipped to support the needs of most elderly citizens, leaving many vulnerable and without the care or income security they require. The analysis in this article highlights how poverty and inadequate pensions trap older Nigerians in financial insecurity, how high costs and limited services restrict their access to healthcare, and how social changes have increased isolation and marginalization of the older adult. These issues, if left unaddressed, will only intensify as the number of older people rapidly grows in the coming decades. However, Nigeria has the opportunity to proactively respond through comprehensive policy reforms. Efforts must focus on expanding health insurance coverage and reducing out‐of‐pocket costs for the older adults, improving rural healthcare infrastructure to serve seniors everywhere, reforming the pension system to provide broad‐based income support in old age, and combating ageism while fostering community support networks for older adults. Implementing and scaling up existing policies—such as fully funding the NSCC's initiatives, rigorously enforcing the National Health Insurance Authority Act, and possibly introducing a universal social pension—are critical next steps. Such reforms are essential for creating a more supportive environment for Nigeria's older adult population. Ensuring that today's and tomorrow's older adults can access affordable, quality healthcare and live with dignity in old age is not just a welfare issue but a mark of societal progress. By taking decisive action now, Nigeria can build a future where aging does not equate to increased vulnerability, but rather where older people are protected, productive, and valued members of the community.

## Author Contributions

**Sunkanmi Folorunsho**: conceptualization, writing – original draft, writing – review and editing, resources.

## Ethics Statement

The author has nothing to report.

## Conflicts of Interest

The author declares no conflicts of interest.

## Data Availability

The author has nothing to report.
